# Supplementary material for: Porous Structural Properties of K or Na-Co Hexacyanoferrates as Efficient Materials for CO2 Capture
Source: Materials (Basel). 2023 Jan 8;16(2):608. doi: 10.3390/ma16020608 (PMC9863694; doi:10.3390/ma16020608)
Supplement: Supplementary file 1 [file materials-16-00608-s001.zip › materials-2061651-supplementary.pdf]

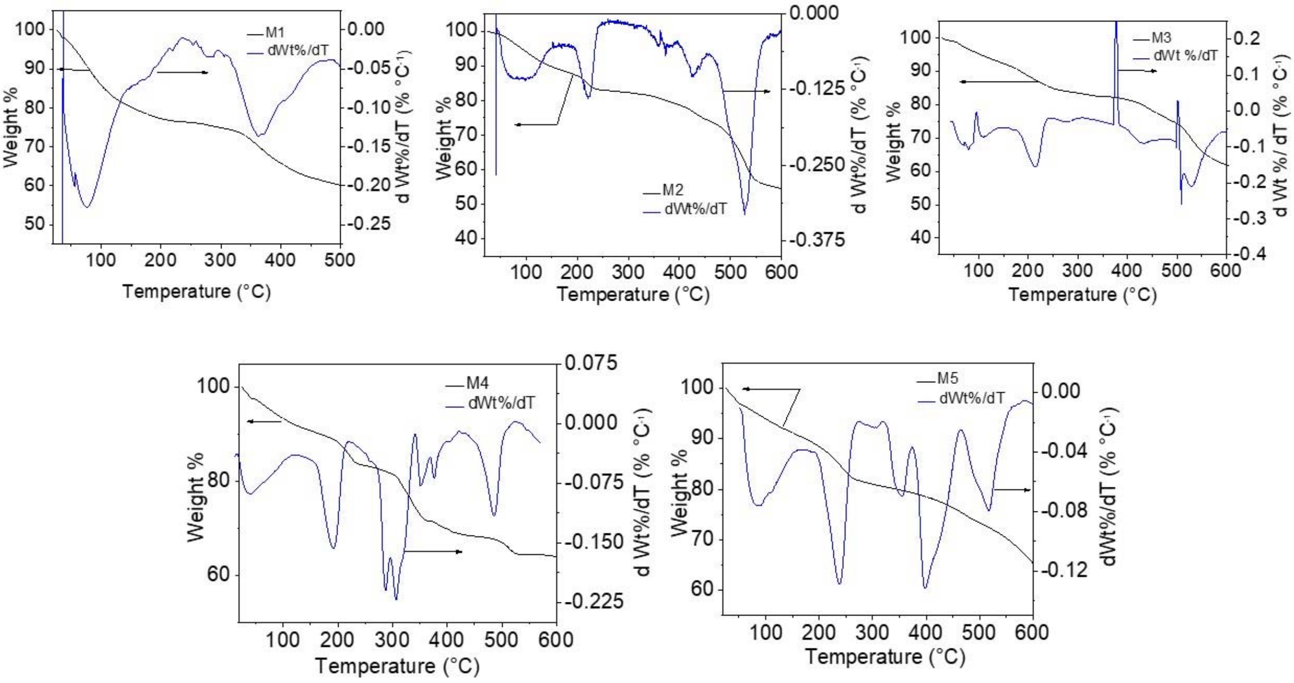

Figure S1. TGA plots (as Weight % loss and first derivative) of samples M1-M5 of CoHCFs.

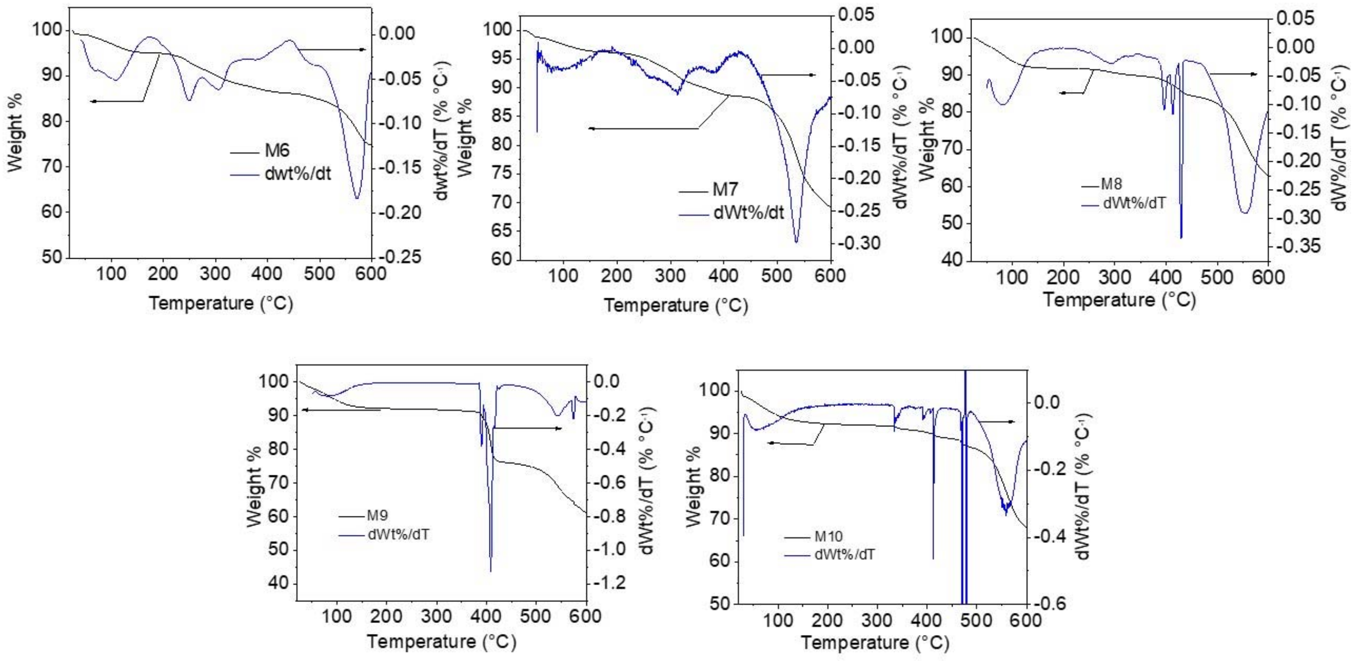

Figure S2. TGA plots (as Weight % loss and first derivative) of samples M6-M10 of CoHCFs.

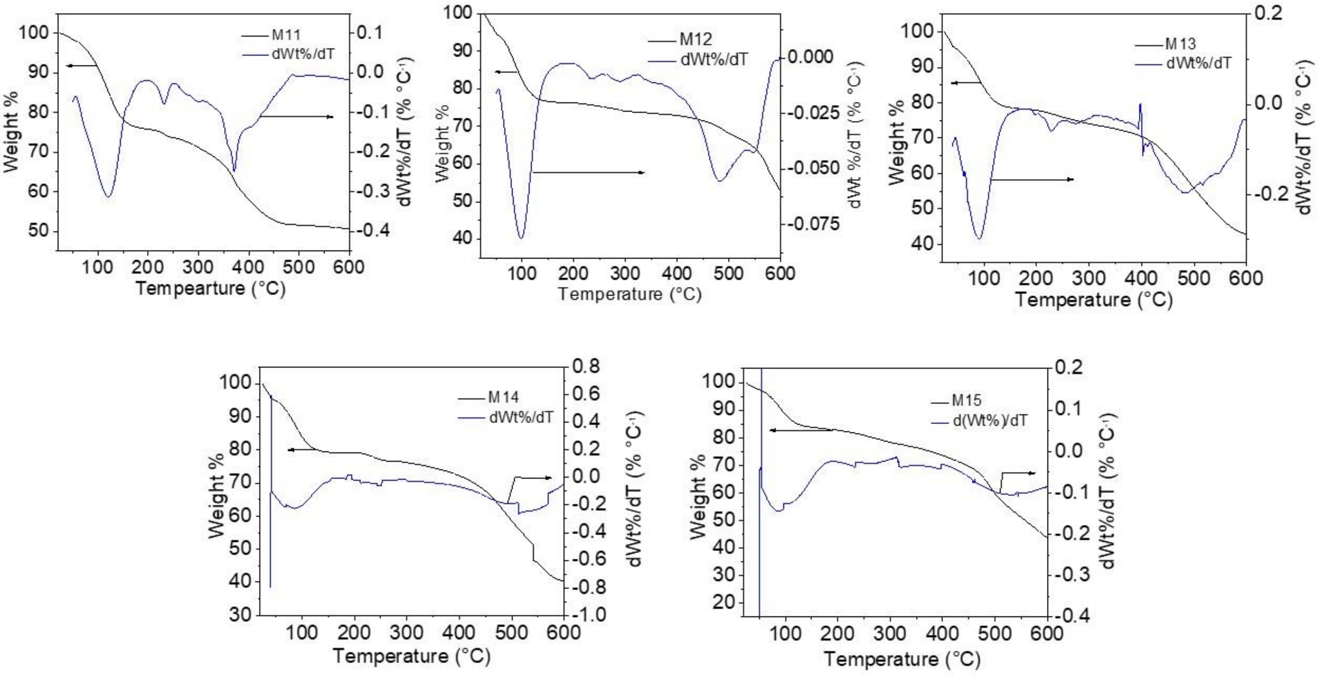

Figure S3. TGA plots (as Weight % loss and first derivative) of samples M11-M15 of CoHCFs.

XRD Rietveld refinement data on sample M6 (identified as JM57).

Refinement final output indices for single spectra:

Datafile Copia de JM57.txt : Rwp: 0.027154693, Rp: 0.020641273, Rwpnb: 0.117103845, Rwpnb1: 0.047824994, Rwpnb2: 0.028283723, Rpnb: 0.1506347Rpnb1: 0.04872347Rpnb2: 0.022067115

Rietveld refinement parameters:

a = 10.056(2) α = 89.96(9)
b = 10.078(2) β = 88.47(2)
c = 10.070(3) γ = 90.39(9)
Rexp (%) = 1.750733

Table S1. XRD Atomic coordinates of sample M6.

ATOM	x	y	z	Biso Factor	Occupancy
K0	0.21339537	0.26930714	0.78935	0.78935	1
K1	0.2496001	0.75391525	0.78258544	0.0046095275	1
K2	0.24240327	0.7701745	0.2682679	0.004609286	1
K3	0.21683615	0.24036963	0.21854377	0.0046096193	1
K4	0.71135676	0.25506827	0.26587352	0.004609263	1
K5	0.70397973	0.756127	0.25189558	0.004609728	1
K6	0.6954931	0.7754539	0.77313817	0.0046092668	1
K7	0.69231606	0.24304779	0.7043417	0.0046095457	1
Fe8	0.007862565	2.6617525×10^{-4}	$-1.7655495 \times 10^{-4}$	0.0046102116	1
Fe9	0.013405314	0.4905588	0.4670046	0.00461002	1

Fe10	0.50426894	0.0016125675	0.47941646	0.0046095983	1
Fe11	0.47650135	0.5025435	−0.0011604204	0.0046108235	1
Co12	0.47473544	0.01544629	0.008525457	0.0046102614	1
Co13	0.49559	0.51086295	0.5130797	0.0046096933	1
Co14	7.144763×10^{-4}	0.013408481	0.5082449	0.0046090684	1
Co15	6.7425007×10^{-4}	0.5076396	0.008664027	0.0046099504	1
C16	0.51991403	0.19398375	−0.0027277668	0.0046093063	1
C17	0.493107	0.5159492	−0.004348543	0.0046094134	1
C18	0.0036056023	7.6885894×10^{-4}	0.8442555	0.0046093212	1
C19	0.77849215	1.5382229×10^{-4}	0.0026891972	0.004609467	1
C20	0.49899247	0.0033021613	0.6528794	0.0046090805	1
C21	0.20132203	0.003101261	9.511535×10^{-4}	0.0046094847	1
C22	0.53107595	0.80303895	0.47454295	0.0046095494	1
C23	0.5035077	0.2395549	0.41985938	0.0046090656	1
C24	0.0037762842	0.4025754	0.26357687	0.0046095867	1
C25	0.8343898	0.53006595	0.51570225	0.004609332	1
C26	0.4736673	0.57889044	0.22773653	0.0046094237	1
C27	0.15110737	0.49002242	0.5022098	0.0046090614	1
C28	0.0052313176	0.21458119	0.45735332	0.004608982	1
C29	2.9247883×10^{-4}	0.7822422	0.26811823	0.0046094405	1
C30	0.47687852	0.0010089532	0.32254997	0.0046087657	1
C31	0.28786176	1.564865×10^{-4}	0.5074434	0.004609527	1

ATOM	x	y	z	Biso Factor	Occupancy
C32	$-9.5801783 \times 10^{-4}$	0.0013666921	0.1868362	0.004609006	1
C33	0.6270393	−0.0017123559	0.51883554	0.004609003	1
C34	0.004478851	0.97109765	−0.009933286	0.004609334	1
C35	3.31533×10^{-4}	0.25316003	−0.0027350863	0.0046091643	1
C36	0.48826376	0.4708269	0.8599329	0.0046095187	1
C37	0.23741168	0.6082885	0.004991114	0.004609309	1
C38	-7.6391×10^{-4}	0.51240784	0.628015	0.0046090637	1
C39	0.6518778	0.483303	4.224629×10^{-4}	0.0046091457	1
N40	0.7013715	0.0079332935	0.0032429472	0.004609518	1
N41	−0.0015269169	0.028602712	0.67092264	0.004609163	1
N42	0.5344147	0.7771988	0.005029325	0.0046091895	1

N43	0.5586271	0.20617616	−0.0044596605	0.0046092677	1
N44	0.4797129	0.001539663	0.8114993	0.0046088947	1
N45	0.28558052	3.3445022×10^{-4}	0.010179597	0.004609405	1
N46	0.7185487	0.5151947	0.48919123	0.0046096263	1
N47	−0.002440368	0.56496763	0.22910292	0.0046093827	1
N48	0.44147006	0.27255192	0.5434033	0.004608775	1
N49	0.48737404	0.61859256	0.4524773	0.00460903	1
N50	0.50129056	0.42573187	0.27916667	0.004609422	1
N51	0.3200124	0.5224988	0.48776066	0.004609317	1
N52	0.22760499	0.0049257716	0.48717323	0.0046098065	1
N53	0.49435413	0.020174203	0.16728099	0.0046090544	1
N54	0.0014522229	0.76633227	0.51911014	0.0046089506	1
N55	0.001489375	0.21474767	0.45598373	0.0046089897	1
N56	0.011848602	0.0013979856	0.29151845	0.0046093957	1
N57	0.8330878	3.1203078×10^{-4}	0.48224202	0.004609227	1
N58	0.22026938	0.45513877	0.004069938	0.0046092514	1
N59	0.50650257	0.52954936	0.6894973	0.0046090363	1
N60	0.0023961428	0.2575178	0.0066635218	0.004609438	1
N61	0.0011668742	0.64178675	−0.0012996352	0.0046094535	1
N62	0.010218649	0.47992063	0.80811375	0.0046089734	1
N63	0.7910187	0.515877	0.0033978862	0.0046094167	1

Table S2. XPS binding energy assignments of samples M1, M6 and M11 of CoHCFs.

Core level	M1			M6			M11		
	B.E. (eV)	Assignment	Ref.	B.E. (eV)	Assignment	Ref.	B.E. (eV)	Assignment	Ref.
C 1s	283.48	CO-Co	[1]	283.55	C–Fe^{2+}	[2]	283.13	Tape	
	283.50	C–Fe^{2+}	[2]	284.47	C(elemental)	[3]	283.77	C–Fe^{2+}	[2]
	284.53	C (elemental)/Co	[3]	285.60	Adventitious	[2]	284.03	C–Fe^{3+}	[2]
	285.4	Adventitious	[2]	287.44	KCN	[2]	285.04	Adventitious	
	287.42	$Co(CN)_x$	Present study						
O 1s	531.4	$Co(NO_3)_2$	[4]	530	CoO	[5]	530.91	Fe_xO_y	[6]
	535.4	H_2O	[7]	531.3	$Co(NO_3)_x$	[8]	531.88	$Co(NO_3)_x$	[9]
				532.58	H_2O	[10]	532.77	H_2O	[5]
				532.3	KNO3	[11]			
N 1s	396.53	N–Co^{2+}	[2]	397.30	N–Co^{2+}	[2]	396.55	Co_xN_y	[2]
	396.95	N–Co^{3+}	[2]	397.76	N–Co^{3+}	[2]	397.21	N–Co^{2+}	[12]
	400.88	$Co(NO_3)_x$	[4]	401.79	$Co(NO_3)_x$	[4]	397.7	N–Co^{3+}	[2]
				406.7	KNO_3	[11]	398.86	Satellite	
Na 1s	1071.34	$Na_nCo^{2+,3+}[Fe(CN)_6]$	[2]						
K 2p	---			292.41	$K_n Co^{2+,3+} [Fe(CN)_6]$	[2]	292.62	$K_n Co^{2+,3+} [Fe(CN)_6]$	[2]
	---			293.55	Satellite		291.74	Satellite	
	---			---			298.71	Satellite	[2]
Co 2p	777.57	Satellite		781.50	Satellite		783.7	Auger Fe	
	780.68	N–Co^{2+}	[4,13]	781.90	N–Co^{2+}	[4,13]	785.54	N–Co^{2+}	[4,13]
	780.80	Satellite		783.58	N–Co^{3+}	[14]	786.36	N–Co^{3+}	[14]
	783.59	N–Co^{3+}	[14]	783.7	Auger Fe		789.01	Satellite	
	783.7	Auger Fe		787.68	Satellite		791.6	Satellite	
	787.03	Satellite					791.69	Satellite	
Fe 2p	707.34	C–Fe^{2+}	[15]	708.230	Fe^0 /Satellite	[16]	711.7	Fe_xO_y	[17]
	712.7	Auger Co		709.230	C–Fe^{2+}	[15]	712.72	C–Fe^{2+}	[15]
	716.54	Satellite		709.490	C–Fe^{2+}	[15]	713.97	Auger Co	
	718.84	Satellite		713.700	Auger Co		715.27	C–Fe^{3+}	[15]
	721.34	Satellite		716.596	Satellite		718.42	Satellite	
	722.57	Satellite		722.145	Satellite		725.15	Satellite	

References

1. D.A. Wesner, G. Linden, H.P. Bonzel, Alkali promotion on cobalt: Surface analysis of the effects of potassium on carbon monoxide adsorption and Fischer-Tropsch reaction, Appl. Surf. Sci. **1986**, *26*, 335–356. https://doi.org/10.1016/0169-4332(86)90074-7.
2. N.G. Vannerberg, The ESCA-spectra of sodium and potassium cyanide and of the sodium and potassium salts of the hexacyanometallates of the first transition metal series., Chem. Scr.**1976**, *9*, 122–126.
3. D.N. Belton, S.J. Schmieg, Electron spectroscopic identification of carbon species formed during diamond growth, J. Vac. Sci. Technol. A Vacuum, Surfaces, Film. **1990**, *8*, 2353–2362. https://doi.org/10.1116/1.576697.
4. C.A. Strydom, H.J. Strydom, X-ray photoelectron spectroscopy studies of some cobalt(II) nitrate complexes, Inorganica Chim. Acta. **1989**, *159*, 191–195. https://doi.org/10.1016/S0020-1693(00)80566-6.
5. V.I. Nefedov, D. Gati, B.F. Dzhurinskii, N.P. Sergushin, Y.V. Salyn, [dataset] A. V. Naumkin, A. Kraut-Vass, S. W. Gaarenstroom, C. J. Powell, NIST X-ray Photoelectron Spectroscopy Database, NIST Standard Reference Database 20, v4.1, 2012., Zhurnal Neorg. Khimii. **1975**, *20*, 2307–2314. https://doi.org/http://dx.doi.org/10.18434/T4T88K.
6. E. Paparazzo, XPS and Auger Spectroscopy on mixtures of the oxides SiO_2, Al_2O_3, Fe_2O_3, and Cr_2O_3, J. Electron Spectros. Relat. Phenomena. **1987**, *43*, 97–112.
7. P.D. Schulze, S.L. Shaffer, R.L. Hance, D.L. Utley, Adsorption of water on rhenium studied by XPS, J. Vac. Sci. Technol. A Vacuum, Surfaces, Film. **1983**, *1*, 97–99. https://doi.org/10.1116/1.572321.
8. J.P. Bonnelle, J. Grimblot, A. D'huysser, Influence de la polarisation des liaisons sur les spectres esca des oxydes de cobalt, J. Electron Spectros. Relat. Phenomena. **1975**, *7*, 151–162. https://doi.org/10.1016/0368-2048(75)80047-8.
9. P. Porta, R. Dragone, G. Fierro, M. Inversi, M. Lo Jacono, G. Moretti, Preparation and characterisation of cobalt-copper hydroxysalts and their oxide products of decomposition, J. Chem. Soc. Faraday Trans. **1992**, *88*, 311–319. https://doi.org/10.1039/FT9928800311.

10. J. Russat, Characterization of Polyamic Acid/Polyimide Films in the Nanometric Thickness Range from Spin-deposited Poly amic Acid, Surf. Interface Anal. **1988**, *11*, 414–420.
11. V.I. Nefedov, Y. V. Salyn, G. Leonhardt, R. Scheibe, A comparison of different spectrometers and charge corrections used in X-ray photoelectron spectroscopy, J. Electron Spectros. Relat. Phenomena. **1977**, *10*, 121–124. https://doi.org/10.1016/0368-2048(77)85010-X.
12. B.M. Biwer, S.L. Bernasek, Electron spectroscopic study of the iron surface and its interaction with oxygen and nitrogen, J. Electron Spectros. Relat. Phenomena. **1986**, *40*, 339–351. https://doi.org/10.1016/0368-2048(86)80044-5.
13. B.J. Tan, K.J. Klabunde, P.M.A. Sherwood, XPS Studies of Solvated Metal Atom Dispersed Catalysts. Evidence for Layered Cobalt—Manganese Particles on Alumina and Silica, J. Am. Chem. Soc. **1991**, *113*, 855–861. https://doi.org/10.1021/ja00003a019.
14. R.O. Lezna, R. Romagnoli, N.R. De Tacconi, K. Rajeshwar, Cobalt hexacyanoferrate: Compound stoichiometry, infrared spectroelectrochemistry, and photoinduced electron transfer, J. Phys. Chem. B. **2002**, *106*, 3612–3621. https://doi.org/10.1021/jp013991r.
15. K.B. Yatsimirskii, V. V. Nemoshkalenko, Y.P. Nazarenko, V.G. Aleshin, V. V. Zhilinskaya, N.A. Tomashevsky, Use of X-ray photoelectron and Mössbauer spectroscopies in the study of iron pentacyanide complexes, J. Electron Spectros. Relat. Phenomena. **1977**, *10*, 239–245. https://doi.org/10.1016/0368-2048(77)85023-8.
16. R. V. Siriwardane, J.M. Cook, Interactions of SO2 with sodium deposited on silica, J. Colloid Interface Sci. **1985**, *108*, 414–422. https://doi.org/10.1016/0021-9797(85)90280-2.
17. K. Kishi, Adsorption of ethylenediamine on clean and oxygen covered Fe/Ni(100) surfaces studied by XPS, J. Electron Spectros. Relat. Phenomena. **1988**, *46*, 237–247. https://doi.org/10.1016/0368-2048(88)80022-7.
